# Age and Sex‐Related Differences in Neuroprotective Effects of Cardiovascular Endurance on Cortical Thickness and Brain Volume in Adults Across Age

**DOI:** 10.1002/brb3.70231

**Published:** 2025-01-19

**Authors:** Junyeon Won, Marissa Gogniat, J. Carson Smith

**Affiliations:** ^1^ Department of Neurology University of Texas Southwestern Medical Center Dallas Texas USA; ^2^ Institute for Exercise and Environmental Medicine Texas Health Presbyterian Hospital Dallas Texas USA; ^3^ Department of Neurology University of Pittsburgh School of Medicine Pittsburgh Pennsylvania USA; ^4^ Department of Kinesiology University of Maryland College Park Maryland USA; ^5^ Program in Neuroscience and Cognitive Science University of Maryland College Park Maryland USA

**Keywords:** aging, brain volume, cortical thickness, fitness, MRI, sex difference

## Abstract

**Background:**

Higher cardiorespiratory fitness and cardiovascular endurance (CE) have been shown to be neuroprotective in older adulthood, but the mechanisms underlying this neuroprotection across the adult lifespan are poorly understood. The current study sought to examine the neuroprotective effects of CRF on gray matter (GM) and white matter (WM) volumes, and mean cortical thickness (MCT), using a large sample across the adult lifespan. We also examined sex differences in these relationships.

**Methods:**

This cross‐sectional study included data from 1691 adults across the adult lifespan (22–100 years) from the Human Connectome Project Young Adults and Aging data. 2‐minute walk test performance was used as a proxy of CE. Structural MRI was used to assess total GM volume, WM volume, and MCT. Linear regression models were used to examine the interaction between age and CE on total GM volume, WM volume, and MCT after adjusting for sex, years of education, body mass index, systolic blood pressure, gait speed, and total intracranial volume. Whole‐brain surface‐based analysis was also performed to investigate regional effects. Finally, interactions between age, sex, and CE were examined to test sex differences. *p* < 0.05, two‐sided test, was designed as statistically significant.

**Results:**

With greater CE, the associations between greater age and lower total and regional GM and WM volumes and MCT were attenuated in whole sample. In men, age was associated with lower total GM volume and MCT regardless of CE level. In women, attenuated negative associations between age and total GM volume and MCT were found in those with high CE.

**Conclusion:**

Greater CE over the adult lifespan is associated with preservation of cortical thickness and brain volume, particularly in women.

## Graphical abstract

This study investigated the moderating effects of greater cardiovascular endurance on the association between aging and gray matter structure in adults across the lifespan. Our findings suggest that higher cardiovascular endurance may attenuate age‐related decline in cortical thickness and brain volume, particularly in women.

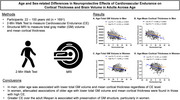

## 1 Introduction

According to the US Census Bureau, the number of people aged 65 and older is rapidly increasing from 54 million in 2020 (16% of population) to 80 million in 2040 (20% of population) (Ortman, Velkoff, and Hogan 2014). With the rising number of older adults comes increased cases of neurodegenerative diseases including Alzheimer's disease (Hou et al. 2019). Changes in brain volume and cortical thickness in areas susceptible to Alzheimer's disease may be early pathological markers for neurodegeneration (Dickerson et al. 2009; Reiter et al. 2017). Specifically, there is a close relationship of aging with brain volume loss and cortical thinning (Dhamoon et al. 2015). Longitudinal studies showed a mean change of −0.3% per year for age‐related cortical thinning across most of the cortices (Shaw et al. 2016) and brain volume decrease at a rate of around 5% per decade after the age of 40 with accelerated brain volume atrophy over the age of 70 (Svennerholm, Boström, and Jungbjer 1997).

Evidence suggests that elements of fitness like cardiorespiratory fitness (measured using VO_2peak_) and cardiovascular endurance (CE; measured via a timed walking test) are related to better brain health outcomes (Won et al. 2024). Intervention studies showed that significantly improved CRF after completion of exercise training was associated with increased regional cortical thickness in older adults (Reiter et al. 2015; Williams et al. 2017). A randomized controlled trial in older adults reported improvements in CRF after a 1‐year exercise and stretching intervention, and improvements in CRF were associated with increases in the cortical thickness of the right inferior parietal lobe (Tarumi et al. 2022). An observational study also demonstrated the link between higher CRF and greater total and regional gray matter (GM) volumes in adults aged between 58 and 81 years (Weinstein et al. 2012). In addition, literature suggests sex‐related differences in the associations between CRF and brain GM volumes (Pentikäinen et al. 2017; Barha et al. 2020; Barha et al. 2019); however, the moderating roles of sex on the association between CRF and brain GM integrity remain inconclusive.

Despite these findings, a major knowledge gap is that most investigations used participants in a similar age range, particularly focused on middle‐aged and older adults. Some evidence suggests that neurodegenerative processes occur in midlife or earlier (Bendlin et al. 2010), thus making this a critical period for further investigation on the effects of CRF on brain structure. Nonetheless, little evidence is available regarding the associations between CRF and brain structure in an adult sample from an adult lifespan perspective. To address this knowledge gap, this cross‐sectional investigation leveraged both Human Connectome Project (HCP) Young Adults and Aging Lifespan data to examine the moderating effects of CE on the associations of age with mean cortical thickness and brain volume in a large and well‐characterized sample of healthy adults across the adult lifespan (22–100 years). In addition, to address the gaps in literature regarding inconclusive sex differences on the associations between CE and brain GM integrity, we assessed the sex‐related differences in the moderating effects of CE on the associations of age with cortical thickness and brain volume using balanced number of women and men samples of the data (Van Essen et al. 2013; Bookheimer et al. 2019). We hypothesized that higher CE may mitigate the deterioration of cortical thickness and brain volume with age and, in women, there will be a greater association between higher CE and lower age‐related decline in cortical thickness and brain volume (Barha et al. 2019).

## 2 Methods

### 2.1 Participants

The Washington University—University of Minnesota Consortium of the Human Connectome Project (WU‐Minn HCP) Young Adults (https://www.humanconnectome.org/) and HCP Aging Lifespan Data (https://www.humanconnectome.org/study/hcp‐lifespan‐aging/data‐releases) were used for the present study (Van Essen et al. 2013; Bookheimer et al. 2019). There was no participant who was enrolled in both studies. Key inclusion criteria included: (1) age 22–35 for Young Adults project, (2) age 36–100+ for Aging Lifespan dataset, and (3) ability to give informed consent. Key exclusion criteria included: (1) significant history of psychiatric disorder, substance abuse, neurological, or cardiovascular disease; (2) two or more seizures after the age of 5 or a diagnosis of epilepsy; and (3) moderate or severe claustrophobia. A complete list of eligibility criteria was previously reported (Van Essen et al. 2013; Bookheimer et al. 2019). At the beginning of their first visit, participants provided written consent approved by the Institutional Review Board of Washington University. The study was completed in accordance with the Declaration of Helsinki. Participants visited Washington University twice for an MRI scan and other assessments including the 2‐min walk test (2MWT). Participants visited the study sites on 2 different days within 2 weeks for MRI and behavioral assessments. The time range of data acquisition and preprocessing for the HCP Young Adults dataset was from 2010 to 2015 and HCP Aging Lifespan dataset was from 2016 to 2021 (Elam et al. 2021).

### 2.2 Demographic Measurements

Age, sex, and education were self‐reported. BMI was calculated based on the self‐reported height and weight (kg/m^2^). Prior to the study visit, participants were asked about hypertension status (≥ 140/90 mmHg) and those who reported the history of hypertension or were taking hypertension medication were excluded. Sitting BP in the left arm was measured once while the arm was relaxed and supported after the participants had rested for at least 5 min.

### 2.3 2‐Minute Walk Test

2MWT was performed as part of the motor domain of the NIH toolbox (Reuben et al. 2013) and was used as a proxy for CE in the present study. Participants walked as fast as they could for 2 min on a 50‐foot course (15.24 m) during the test. The total distance walked in 2 min was measured in feet and inches and used as raw scores. Next, raw scores were normalized to the normative sample (≥ 18 years old) in the NIH Toolbox, regardless of age, sex, and other variables. The scores were normalized to have a mean of 100 with standard deviation of 15. Higher 2MWT scores are associated with longer walking distance and thus, better performance.

### 2.4 Gait Speed Measurement

The 4‐meter gait test, administered as a part of the motor assessment of the NIH toolbox (Reuben et al. 2013), is an established and valid measurement of gait speed. Participants walked 4 meters at their usual pace during the test. After completing a practice session, participants performed two timed 4‐meter walking tests and the best trial was used for scoring. The test score was measured as meters per second (m/s). Previous research has demonstrated increased gait variability in older adults during 2‐min walking tests (Ortman, Velkoff, and Hogan 2014). Thus, we used gait speed as a covariate in the regression models to factor in variance in 2MWT performance across different age that could be attributed to differences in gait speed.

### 2.5 MRI Data Acquisition and Processing

Whole‐brain magnetic resonance imaging (MRI) was conducted using a customized Siemens (Munich, Germany) 3.0 Tesla Skyra MR scanner at Washington University. A 32‐channel head coil was used for radio frequency transmission and reception. For the HCP Young Adults data, a high‐resolution T1‐weighted anatomical image was acquired with gradient echo sequence: field of view = 224 mm, voxel size = 0.7 × 0.7 × 0.7 mm, slice thickness = 0.9 mm, repetition time = 2400 ms, echo time = 2.14 ms, inversion time = 1000 ms, flip angle = 8°, and duration = 7:40 min. For the HCP Aging Lifespan Data, a high‐resolution T1‐weighted anatomical image was acquired with gradient echo sequence: field of view = 256 mm, voxel size = 0.8 × 0.8 × 0.8 mm, slice thickness = 0.8 mm, repetition time = 2500 ms, echo time = 1.81 ms, inversion time = 1000 ms, flip angle = 8°, and duration = 8:22 min. Participants' structural MRI data were entered into FreeSurfer's (version 5.2.0; http://surfer.nmr.mgh.harvard.edu) fully automated cortical parcellation and subcortical segmentation pipelines (recon‐all) (Fischl 2012), calculating volumes of cortical and subcortical GM, WM structures, total intracranial volume (ICV), and cortical thickness (Fischl et al. 2004). We performed a review of individual image data to ensure data processing quality.

### 2.6 Statistical Analysis

First, we investigated the associations of age or 2MWT with total brain volume and mean cortical thickness using linear regression models. Age (continuous variable) or 2MWT was added as an independent continuous variable, and total GM volume, total WM volume, and mean cortical thickness were, respectively, included as dependent variables in separate models. Covariates included sex, years of education, body mass index (BMI), systolic blood pressure (SBP), and gait speed. ICV was further included as a covariate for regression models on brain volume. We also accounted for the differences in MRI data acquisition between HCP projects in the statistical models to adjust for potential confounding effects; however, the MRI protocol was not related to the MRI outcomes. Benjamini–Hochberg false discovery rate (FDR) correction was conducted to control the family‐wise error rate for multiple comparisons (Benjamini and Hochberg 1995). Next, we tested the associations of age × 2MWT interaction with brain volume and mean cortical thickness using regression models that were adjusted for age, 2MWT score, sex, years of education, BMI, SBP, and gait speed. ICV was further adjusted for regression models for brain volume. Finally, age × 2MWT × sex interaction was further included in the regression model to investigate the possible sex differences. For the statistically significant age × 2MWT × sex interaction findings, the sample was divided into lower and higher fitness groups using the median split of 2MWT performance. We subsequently created regression slopes of age with total brain volume and mean cortical thickness measurements, which were then created for men and women separately to understand the direction of the findings. We used the Shapiro–Wilk test and QQ plots were to assess the normality of the residuals of the model fit to ensure that the model assumptions were met. All statistical analyses were performed using SPSS (v. 26.0, IBM, Armonk, NY) with two sided tests, *p* < 0.05.

### 2.7 Whole‐Brain Surface‐Based Analyses

To further understand the interaction effects between age and 2MWT at the regional level, group‐level surface area and cortical thickness analyses were conducted for both left and right hemispheres using FreeSurfer's whole‐brain surface‐based group analysis tool (Fischl 2012). Within this approach, multiple regression analyses were performed at each vertex along the cortical mesh. First, surface data were smoothed with a 10 mm full‐width at half maximum kernel in preparation for group analyses (mris_preproc). Second, general linear model analyses were performed in the cortical thickness and surface area for both left and right hemispheres (mri_glmfit). In the first regression model, age (continuous variable) or 2MWT was added as an independent variable. Dependent variables were vertex‐wise thickness, cortical thickness, and surface area. Covariates included sex, years of education, BMI, gait speed, and SBP. ICV was further adjusted for the regression models on surface area. In the second regression model, age and 2MWT interaction was further included to identify brain voxels associated with age × 2MWT. Lastly, age × 2MWT × sex interaction was further included into the regression model to identify sex‐related differences. To control the family‐wise error rate across both hemispheres, we administered cluster‐based correction for multiple comparisons using FreeSurfer's permutation simulation approach (mri_glmfit‐sim) with a vertex‐wise threshold of *p* < 0.001 and cluster threshold of *p* < 0.05 (Greve and Fischl 2018).

## 3 Results

### 3.1 Participants

Among a total of 1931 participants who completed the study protocol (combining HCP Young Adult and Aging Lifespan Data), 93 individuals were excluded due to uncollected MRI data, 133 participants were excluded due to missing 2MWT results, and 14 participants were excluded due to missing BP data. A complete description of participant demographic information is presented in Table 1. Age and SBP gradually increased from young age group to older age group. 2MWT gradually decreased from young age group to older age group. DBP increased from young age group to middle‐age group but decreased in older age group. Middle‐aged group had greater BMI compared to young age and older age groups.

**TABLE 1:** Demographic data of the participants.

	Total sample (*n* = 1691)	Young (*n* = 1153)	Middle‐age (*n* = 272)	Older (*n* = 266)	Age group difference
	Mean (SD)	Mean (SD)	Mean (SD)	Mean (SD)	
Age (years)	39.2 (16.9)	29.2 (4.0)	49.2 (5.9)	72.3 (9.0)	< 0.0001
Female (*n*, %)	935 (55.2%)	625 (54.2%)	163 (59.9%)	147 (55.2%)	0.233_F_
Education (years)	15.8 (2.2)	15.0 (1.8)	17.3 (2.2)	17.6 (2.1)	< 0.0001
Body mass index (kg/m^2^)	26.6 (5.0)	26.4 (5.0)	27.7 (5.0)	26.3 (4.5)	0.001
Systolic blood pressure (mmHg)	126.2 (15.8)	123.5 (14.2)	126.9 (15.7)	137.3 (17.5)	< 0.0001
Diastolic blood pressure (mmHg)	78.3 (11.0)	76.5 (10.7)	82.3 (11.0)	82.5 (10.0)	< 0.0001
Gait speed (m/s)	1.30 (0.2)	1.31 (0.2)	1.30 (0.2)	1.2 (0.2)	< 0.0001
2‐min walk test	106.4 (13.4)	109.9 (11.9)	103.2 (10.9)	94.5 (14.1)	< 0.0001
Race (*n*, %)					
White	1244 (73.5%)	850 (73.7%)	168 (61.7%)	226 (84.9%)	
Black or African American	248 (14.6%)	174 (15.0%)	53 (19.4%)	21 (7.8%)	
Asian/Native Hawaiian/Other Pacific Islander	109 (6.4%)	71 (6.1%)	22 (8.0%)	16 (6.0%)	
American Indian/Alaskan Native	3 (0.1%)	2 (0.1%)	1 (0.3%)	0 (0%)	
More than one	55 (3.2%)	35 (3.0%)	17 (6.2%)	3 (1.1%)	
Unknown or not reported	32 (1.8%)	21 (1.8%)	11 (4.0%)	0 (0%)	

*Note*: Young, 22–39 years old; middle‐age, 40–59 years old; older, 60–100 years old; 2‐min walk test scores were normalized across all participants in the study to have a mean of 100 with standard deviation of 15, indicating 100 reflects the national average performance and scores of 85 and 115, respectively, reflect performances 1 SD below and above the national average. Higher scores indicate longer walk distance. Group difference (*p* value) was calculated using one‐way ANOVA with young group as a reference group.

Abbreviations: F, Fisher's exact test; SD, standard deviation.

### 3.2 Associations of Age and 2MWT With Total Brain Volume and Mean Cortical Thickness

After adjusting for sex, years of education, BMI, SBP, gait speed, and estimate ICV, older age was significantly associated with lower total GM volume, total WM volume, and mean cortical thickness. Higher 2MWT score was associated with greater total GM volume, total WM volume, and mean cortical thickness after adjusting for sex, years of education, BMI, SBP, gait speed, and ICV (Table 2).

**TABLE 2:** Associations of age and 2‐min walk test score with total gray matter volume, total white matter volume, and mean cortical thickness.

	Age				2MWT			
	*β*	SE	*p* value	95% CI	*β*	SE	*p* value	95% CI
Total gray matter volume	−1976.859	63.023	1.94E‐170	−2099.791, −1852.448	598.774	82.060	4.52E‐13	437.824, 759.725
Total white matter volume	−673.641	60.014	3.02E‐28	−791.286, −555.813	268.333	79.078	0.0007	113.229, 423.436
Mean cortical thickness	−0.003	0.0001	8.85E‐100	−0.004, −0.003	0.0005	0.0002	0.006	0.0001, 0.0010

*Note*: 2MWT, 2‐min walk test; *β*, unstandardized beta coefficient; SE, unstandardized coefficient standard error; *p* values are corrected for false discovery rate; 95% CI, 95% confidence interval (min and max); Regression models for age were adjusted for sex, years of education, BMI, systolic blood pressure, gait speed, and total intracranial volume; Regression models for 2MWT were adjusted for age, sex, years of education, BMI, systolic blood pressure, gait speed, and total intracranial volume.

### 3.3 Interaction between Age, Sex, and 2MWT on Total Brain Volume and Mean Cortical Thickness

After adjusting for age, 2MWT, sex, years of education, BMI, SBP, gait speed, and ICV, the interaction between age and 2MWT was positively associated with greater total GM volume (*β* = 9.080, *p* = 0.010, 95% CI = 2.167, 15.993), greater total WM volume (*β* = 21.561, *p* = 1.87E‐10, 95% CI = 14.964, 28.156), and greater mean cortical thickness (*β* = 0.00005, *p* = 4.08E‐08, 95% CI = 0.00003, 0.00007). These effects indicated that the associations between greater age and reduced total GM volume, total WM volume, and mean cortical thickness were attenuated by 9.080 mm^3^, 21.561 mm^3^, and 0.00005 mm, respectively, with each 1‐unit increase in 2MWT performance.

**FIGURE 1:**
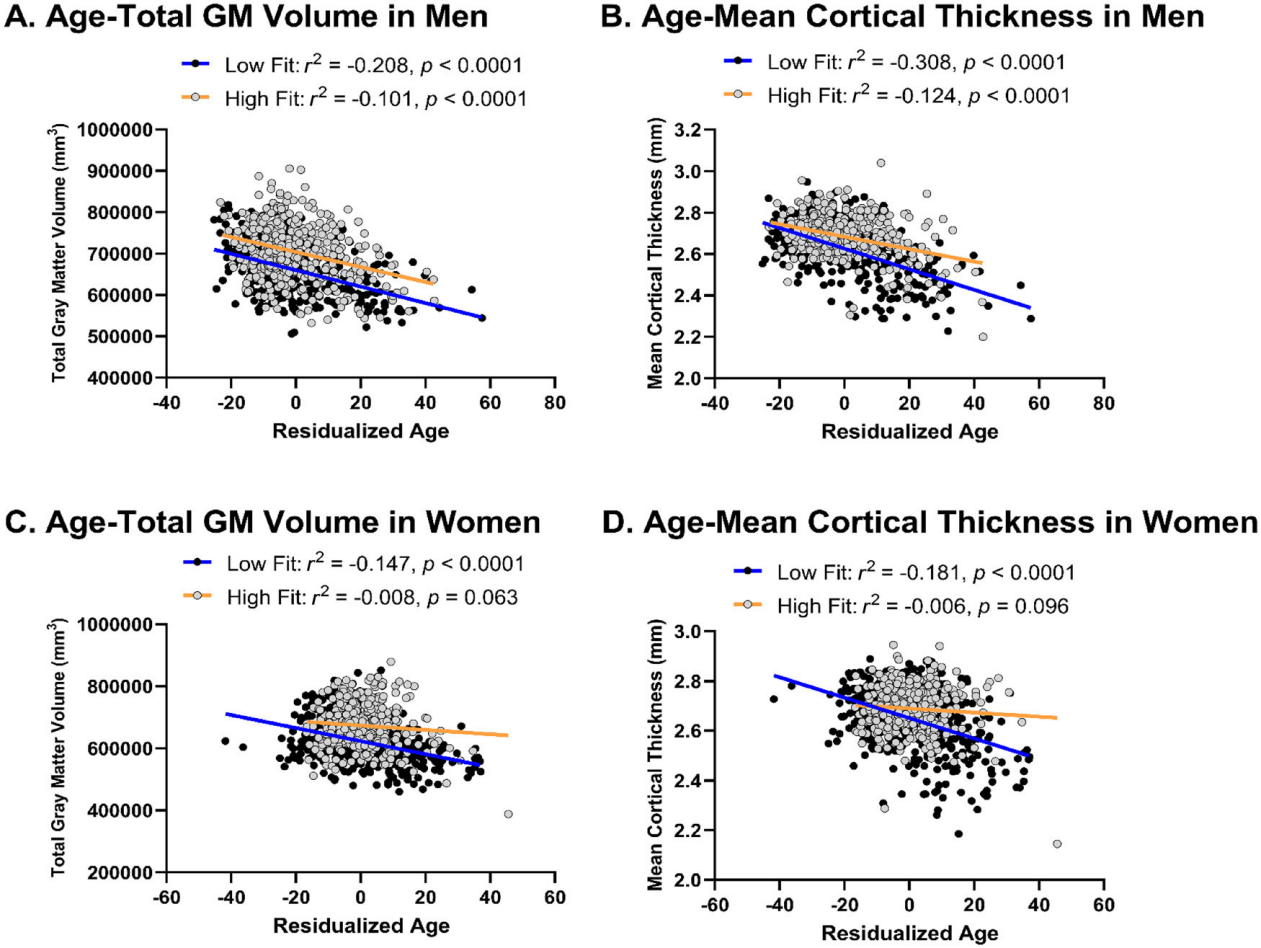
Age × sex × 2MWT interaction plot depicting the regression line of total gray matter volume and mean cortical thickness on age at median split of 2MWT (high fit vs. low fit) in men and women. (A) Association between age and total gray matter volume in men. (B) Association between age and mean cortical thickness in men. (C) Association between age and total gray matter volume in women. (D) Association between age and mean cortical thickness in women.

We further investigated sex‐related differences in the interaction between age and 2MWT on total brain volume and mean cortical thickness. We found significant age × sex × 2MWT interactions on total GM volume (*p* = 0.003, 95% CI = −2.989, −0.604) and mean cortical thickness (*p* = 0.0001, 95% CI = −9.38E‐06, −3.03E‐06). To further understand these interaction effects, we divided the participants into lower and higher fitness using 2MWT score median split. There were no significant associations between age and total GM volume and mean cortical thickness in higher fit women, with age explaining 0.8% of total GM volume (*r* ^2^ = −0.008, *p* = 0.063), while older age was associated with lower total GM volume in women with lower fit women, with age explaining 14.7% of total GM volume (*r* ^2^ = −0.147, *p* < 0.0001). Similarly, no significant association between age and mean cortical thickness was observed in higher fit women, with age explaining 0.6% of mean cortical thickness (*r* ^2^ = −0.006, *p* = 0.096). Conversely, in lower fit women, older age was significantly associated with lower mean cortical thickness with age explaining 18.1% of mean cortical thickness (*r* ^2^ = −0.181, *p* < 0.0001). In men, older age was associated with lower total GM volume in both fit groups, with age explaining 20.8% of total GM volume (*r* ^2^ = −0.208, *p* < 0.0001) in lower fit group and 10.1% of total GM volume (*r* ^2^ = −0.101, *p* < 0.0001) in higher fit group. In addition, in men, older age was associated with lower total mean cortical thickness in both fit groups, with age explaining 30.8% of total GM volume (*r* ^2^ = −0.308, *p* < 0.0001) in lower fit group and 12.4% of total GM volume (*r* ^2^ = −0.124, *p* < 0.0001) in higher fit group (Figure 1). No age × sex × 2MWT interaction was found in total WM volume (*p* = 0.700, 95% CI = –1.378, 0.925).

### 3.4 Associations of Age and 2MWT With Surface‐Based Cortical Volume and Thickness

Widespread clusters were found for the association between older age and lower cortical thickness. Peak regions of the significant clusters include the left rostral middle frontal gyrus (–22.1, 41.5, 24.1 [LPI]; 60,737.87 mm^2^) and right superior parietal lobule (30.6, −47.5, 44.4 [LPI]; 66,220.73 mm^2^). On the other hand, the association between greater age and higher cortical thickness was also observed. Peak regions of the significant clusters include the left pericalcarine (−12.8, −89.0, 0.1 [LPI]; 2289.25 mm^2^), left insula (−34.5, 4.0, −14.9 [LPI]; 968.86 mm^2^), right insula (36.1, −3.4, −4.3 [LPI]; 850.37 mm^2^), and right pericalcarine (15.2, −89.6, 3.0 [LPI]; 792.93 mm^2^) (Figure S1, Panel A).

Similarly, widespread clusters were observed for the association between greater age and lower cortical volume in the left rostral middle frontal gyrus (−22.1, 41.5, 24.1 [LPI]; 60,737.87 mm^2^) and right superior parietal lobule (30.6, −47.5, 44.4 [LPI]; 66,220.73 mm^2^). Meanwhile, the association between greater age and higher cortical volume was also observed in the left pericalcarine (−12.8, −89.0, 0.1 [LPI]; 2289.25 mm^2^), left insula (−34.5, 4.0, −14.9 [LPI]; 968.86 mm^2^), right insula (36.1, −3.4, −4.3 [LPI]; 850.37 mm^2^), and right pericalcarine (15.2, −89.6, 3.0 [LPI]; 792.93 mm^2^) (Figure S1, Panel B).

**FIGURE 2:**
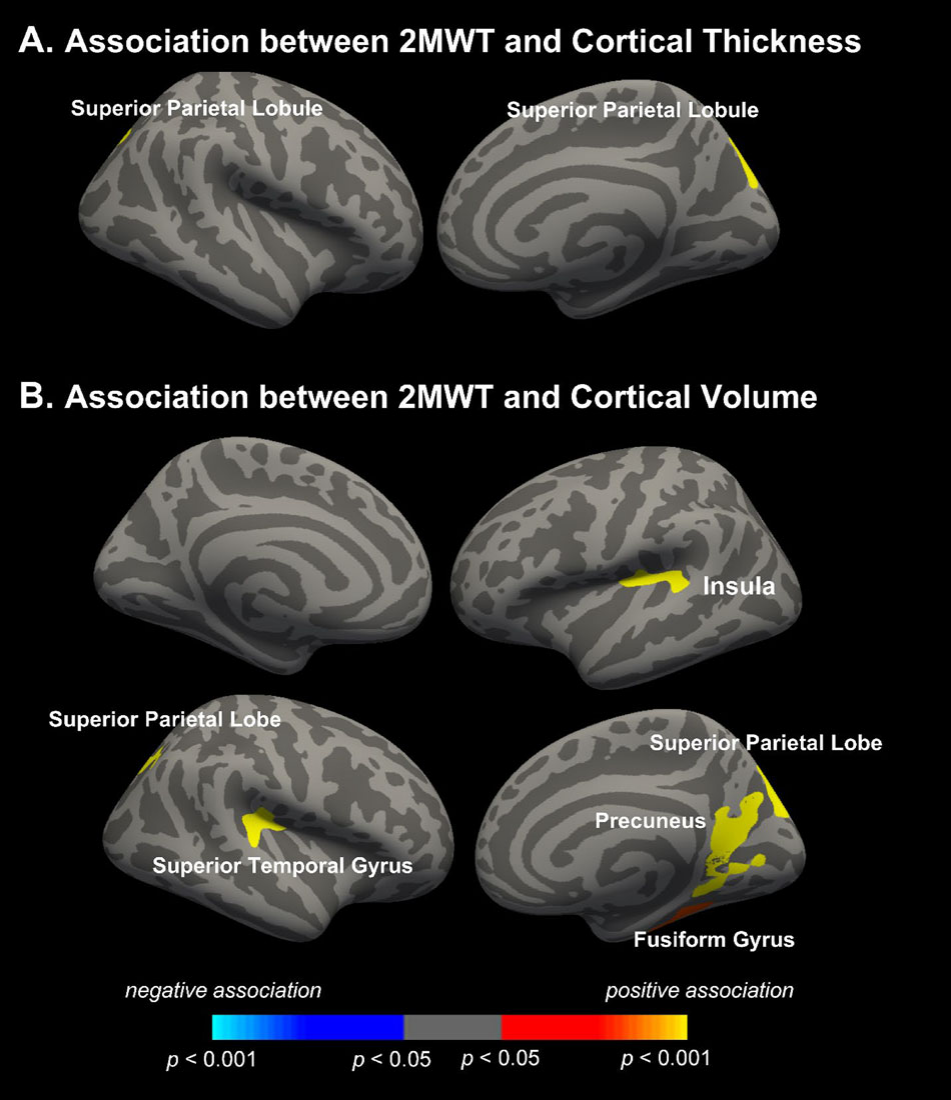
Whole brain surface‐based analysis results of the associations of 2MWT with regional (A) cortical thickness and (B) cortical volume. Clusters survived after correction for multiple comparisons (vertex *p* < 0.001, cluster *p* < 0.05). Warm colors indicate positive association and cool colors indicate negative association.

Better 2MWT performance was associated with greater cortical thickness within the left superior parietal lobule (17.5, −83.9, 38.2 [LPI]; 792.55 mm^2^) (Figure 2, Panel A). In addition, better 2MWT performance was associated with greater surface areas within the left insula (−32.0, −24.5, 16.7 [LPI]; 588.52 mm^2^), right precuneus (18.2, −55.2, 16.4 [LPI]; 1709.83 mm^2^), right superior parietal lobule (15.3, −84.4, 37.4 [LPI]; 1284.32 mm^2^), right superior temporal gyrus (45.4, −31.8, 9.1 [LPI]; 618.86 mm^2^), and right fusiform gyrus (34.9, −39.9, −17.5 [LPI]; 456.20 mm^2^) (Figure 2, Panel B).

### 3.5 Interactions Between Age and 2MWT on Whole‐Brain Surface‐Based Cortical Thickness and Volume

**FIGURE 3:**
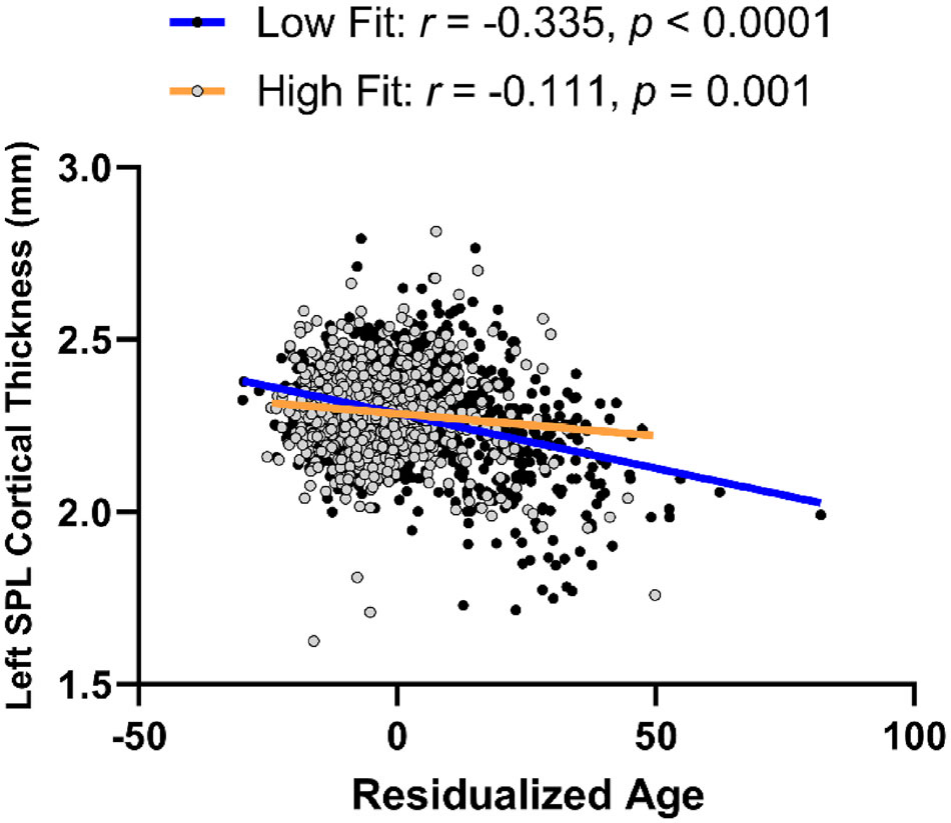
Age × 2MWT interaction plot depicting the regression line of the left superior parietal lobule cortical thickness at median split of 2MWT (high fit vs. low fit). SPL, superior parietal lobule.

There were interactions between age and 2MWT on regional cortical thickness and surface area such that the interaction between age and 2MWT was positively associated with greater left superior parietal lobule cortical thickness (*β* = 0.00006, SE = 0.00001, *p* = 0.00001, 95% CI = 0.00003, 0.00009) (Figure 3). However, we did not observe interaction between age, sex, and 2MWT on left superior parietal lobule cortical thickness (*p* = 0.089).

We did not observe any interaction between age and 2MWT on regional surface areas including the left insula (*p* = 0.104, 95% CI = −0.009, 0.095), right precuneus (*p* = 0.711, 95% CI = −0.078, 0.114), right superior temporal area (*p* = 0.128, 95% CI = −0.008, 0.156), right superior parietal (*p* = 0.417, 95% CI = −0.073, 0.177), and right fusiform gyrus (*p* = 0.826, 95% CI = −0.088, 0.070).

## 4 Discussion

Using a sample of adults across lifespan, the present study provides evidence that (1) higher 2MWT performance attenuated negative associations of age with total and regional brain volume and mean cortical thickness and (2) the attenuated negative associations of age with total GM volume and mean cortical thickness with higher 2MWT performance were observed in women, but not in men. These findings, collectively, suggest that high CE might mitigate the brain structural atrophy across age and those effects are more evident in women than in men.

Consistent with our hypothesis and prior literature, aging was associated with lower total WM volume, total GM volume, and mean cortical thickness (Hafkemeijer et al. 2014; de Chastelaine et al. 2019). In agreement with prior literature on normal aging, our findings suggest that brain atrophy is observed globally. Interestingly, irrespective of age, higher 2MWT performance was associated with greater total WM volume, GM volume, and mean cortical thickness. While higher physical activity level and CRF have been previously shown to be associated with preserved structural brain health in older (Koblinsky et al. 2021) and middle‐aged samples (Castells‐Sánchez et al. 2021), there has been little investigation across the adult lifespan. Our results expand upon prior research by using a large cohort of adults spanning the second to the tenth decade of life and suggest that CE may be protective across young to older adulthood. Although the exact neurobiological mechanisms underpinning the neuroprotective effects induced by higher CE remain equivocal, possible mechanisms include protection of neurovascular unit (Soto et al. 2015), promotion of glymphatic clearance (He et al. 2017), upregulation of brain‐derived neurotrophic factor (BDNF) (Griffin et al. 2011), and neural growth factor (Ang et al. 2003).

The associations between age and cortical volume in regions of interest showed mixed findings. Older age was associated with lower cortical thickness in frontal and superior parietal regions, in line with our hypotheses and prior work (McGinnis et al. 2011); however, older age was also associated with greater cortical thickness in the insula and right pericalcarine regions. Some evidence has shown age‐related increases in cortical thickness in frontal, temporal, and parietal regions (Dotson et al. 2016) due to age‐related inflammatory processes that differentially increase thickness (Dotson et al. 2016). The mechanism underlying the positive associations between age and thickness in the insula and right pericalcarine regions remains unclear so that more studies are required to clarify this research question. Greater CE was also associated with greater cortical thickness in the superior parietal lobe and greater cortical volume mostly in the parietal and some temporal lobe regions of interest, particularly in the left superior parietal lobe. This finding is partially consistent with a study that showed greater functional activation within the superior parietal cortex during working memory task in aerobically trained older adults compared to nontrained counterparts (Colcombe et al. 2004).

Interestingly, we observed sex differences in the interaction between age and CE on total GM volume and mean cortical thickness. Specifically, in high fit women, there were no associations between age and total GM volume and mean cortical thickness, while greater age was associated with both lower total GM volume and mean cortical thickness in lower fit women. Meanwhile, greater age was associated with lower GM volume and cortical thinning in both high fit and low fit men. Our findings of the CE‐related benefits on the brain preferentially in women are in line with previous findings including a meta‐analysis (Colcombe and Kramer 2003), longitudinal study (Barha et al. 2020), and randomized controlled trial (Varma et al. 2015). These findings, however, mainly used older adults and the present study extends these results by using a large cohort of adult sample across age. The sex‐specific effects of CE on the brain are presumably attributed to multiple factors including sex hormones‐related differences in brain structure (Barha et al. 2017) and neuromodulators (e.g., BDNF; Chan and Ye 2017) as well as sex differences in exercise capacity and physiological adaptations to exercise (Barha and Liu‐Ambrose 2018). Nevertheless, the underlying neurobiology remains speculative due to a small number of studies, warranting further investigations in the future. Despite these preferential benefits induced by CE on women, some studies in young and older men demonstrated beneficial impacts of exercise on cerebral blood flow and functional connectivity in men (Kleinloog et al. 2019; Raichlen et al. 2016), suggesting the benefits for men could be reflected in other measures of brain structure or function that were not reported here.

### 4.1 Strengths and Limitations

A major strength of the present study is the large and well‐characterized HCP sample across adult lifespan. Another strength is rich demographic and lifestyle information which allowed us to adjust for many potential confounding factors such as BMI, SBP, and gait speed when examining the associations of age and CE with brain morphological differences. The high‐resolution MRI data collected in the HCP also led to reliable observations. Finally, thanks to the almost equal distribution of sex (54.3% women), we were able to examine sex‐specific effects. A major limitation of the present study is that the sample predominately consisted of young adults due to a disproportionately large number of young adults from HCP young adult sample. Our sample was also well educated overall, which may limit generalizability. However, finding age‐related associations in a predominantly young adult sample provides further credibility to these results. A second limitation is the cross‐sectional design, which precludes any causal interpretations of the data presented. Next, we used 2MWT as a proxy of CE. Although we statistically adjusted variables that may confound health and 2MWT performance in the regression models, 2MWT performance does not perfectly reflect a gold‐standard measure of CRF. Thus, future investigations should determine if oxygen consumption during a maximal graded exercise test is related to 2MWT performance. Finally, generally healthy individuals were recruited for the study which may limit the generalizability of the results for the general population. Future longitudinal studies with a greater diversity in health status are necessary to confirm the findings in the present study.

## 5 Conclusions

Using a well‐characterized and large cohort of younger to older adults, the present study provides evidence that, with advanced age, there are greater neuroprotective effects related to higher CE on total and regional brain volume and cortical thickness. Importantly, these neuroprotective effects on brain structural measurements were evident in women, but not in men. In conclusion, the present study suggests that maintaining or improving cardiovascular fitness may be an important strategy to protect against age‐related brain atrophy among older women. These findings have clinical implications given that age‐related GM atrophy co‐occurs with cognitive decline (Ramanoël et al. 2018) and is commonly observed in neurodegenerative diseases including Alzheimer's disease and related dementias (Anderson et al. 2012). Clinicians may consider prescribing activities that will preserve or improve CRF. Future work is warranted to better understand the mechanisms responsible for sex differences in the associations between CE and healthy brain aging.

## Author Contributions

**Junyeon Won**: conceptualization, investigation, writing–original draft, methodology, validation, visualization, writing–review and editing, formal analysis, data curation. **Marissa Gogniat**: writing–review and editing, methodology. **J. Carson Smith**: writing–review and editing, supervision, data curation, methodology.

## Ethics Statement

Participants provided written consent approved by the Institutional Review Board of Washington University. The study was completed in accordance with Helsinki Declaration.

## Conflicts of Interest

The authors declare no conflicts of interest.

### Peer Review

The peer review history for this article is available at https://publons.com/publon/10.1002/brb3.70231.

## Supporting information



Table S1. Demographic data of the participants stratified by sex.
**Figure S1**. Whole brain surface‐based analysis results of the associations of age with regional (A) cortical thickness and (B) cortical volume. Clusters survived after correction for multiple comparisons (vertex *p* < 0.001, cluster *p* < 0.05). Warm colors indicate positive association and cool colors indicate negative association.

## Data Availability

Data of this study were provided by the Human Connectome Project, WU–Minn Consortium (Principal Investigators: David Van Essen and Kamil Ugurbil; 1U54MH091657) and was funded by the 16 NIH Institutes and Centers that support the NIH Blueprint for Neuroscience Research; and by the McDonnell Center for Systems Neuroscience at Washington University. The data that support the findings of this study are openly available in HCP at https://db.humanconnectome.org/app/template/Login.vm.
