# Supplementary material for: Age and Sex‐Related Differences in Neuroprotective Effects of Cardiovascular Endurance on Cortical Thickness and Brain Volume in Adults Across Age
Source: Brain Behav. 2025 Jan 19;15(1):e70231. doi: 10.1002/brb3.70231 (PMC11743984; doi:10.1002/brb3.70231)
Supplement: Supplementary file 1 — Table S1. Demographic data of the participants stratified by sex. Figure S1. Whole brain surface‐based analysis results of the associations of age with regional (A) cortical thickness and (B) cortical volume. Clusters survived after correction for multiple comparisons (vertex p < 0.001, cluster p < 0.05). Warm colors indicate positive association and cool colors indicate negative association. [file BRB3-15-e70231-s001.docx]

Supplementary Table 1. Demographics data of the participants stratified by sex.

|  | Young (n = 1153) | | Middle-Age (n = 272) | | Older (n = 266) | |
| --- | --- | --- | --- | --- | --- | --- |
|  | Male (n = 528) | Female (n = 625) | Male (n = 109) | Female (n = 163) | Male (n = 119) | Female (n = 147) |
| Age (years) | 28.3 (4.0) | 29.9 (3.8) | 49.4 (5.9) | 49.0 (5.9) | 71.9 (8.1) | 72.6 (9.6) |
| Education (years) | 14.9 (1.9) | 15.1 (1.8) | 17.5 (2.1) | 17.2 (2.2) | 17.8 (2.1) | 17.5 (2.0) |
| Body Mass Index (kg/m^2^) | 27.9 (5.1) | 25.1 (4.6) | 27.7 (3.9) | 27.6 (5.6) | 27.0 (3.7) | 25.7 (4.9) |
| Systolic Blood Pressure (mmHg) | 135.4 (10.4) | 113.5 (8.0) | 128.7 (15.0) | 125.7 (16.1) | 138.3 (18.4) | 136.5 (16.9) |
| Diastolic Blood Pressure (mmHg) | 82.9 (9.5) | 71.0 (8.2) | 84.3 (11.0) | 80.9 (10.9) | 84.7 (10.5) | 80.7 (9.2) |
| Gait Speed (m/s) | 1.31 (0.21) | 1.31 (0.20) | 1.32 (0.24) | 1.30 (0.23) | 1.25 (0.22) | 1.19 (0.24) |
| 2-Minute Walk Test | 110.8 (12.4) | 109.2 (11.3) | 106.8 (10.9) | 100.8 (10.1) | 99.6 (14.0) | 90.4 (12.8) |
| Notes: SD, standard deviation; Young, 22-39 years old; Middle-Age, 40-59 years old; Older, 60-100 years old; 2 Minute Walk Test score were normalized across all participants in the study to have a mean of 100 with standard deviation of 15, indicating 100 reflects the national average performance and scores of 85 and 115, respectively, reflect performances 1 SD below and above the national average. Higher scores indicate longer walk distance. | | | | | | |

**Supplementary Fig 1.** Whole brain surface-based analysis results of the associations of age with regional (a) cortical thickness and (b) cortical volume. Clusters survived after correction for multiple comparisons (vertex *p* < 0.001, cluster *p* < 0.05). Warm colors indicate positive association and cool colors indicate negative association.

**Supplementary Fig 1.**

**
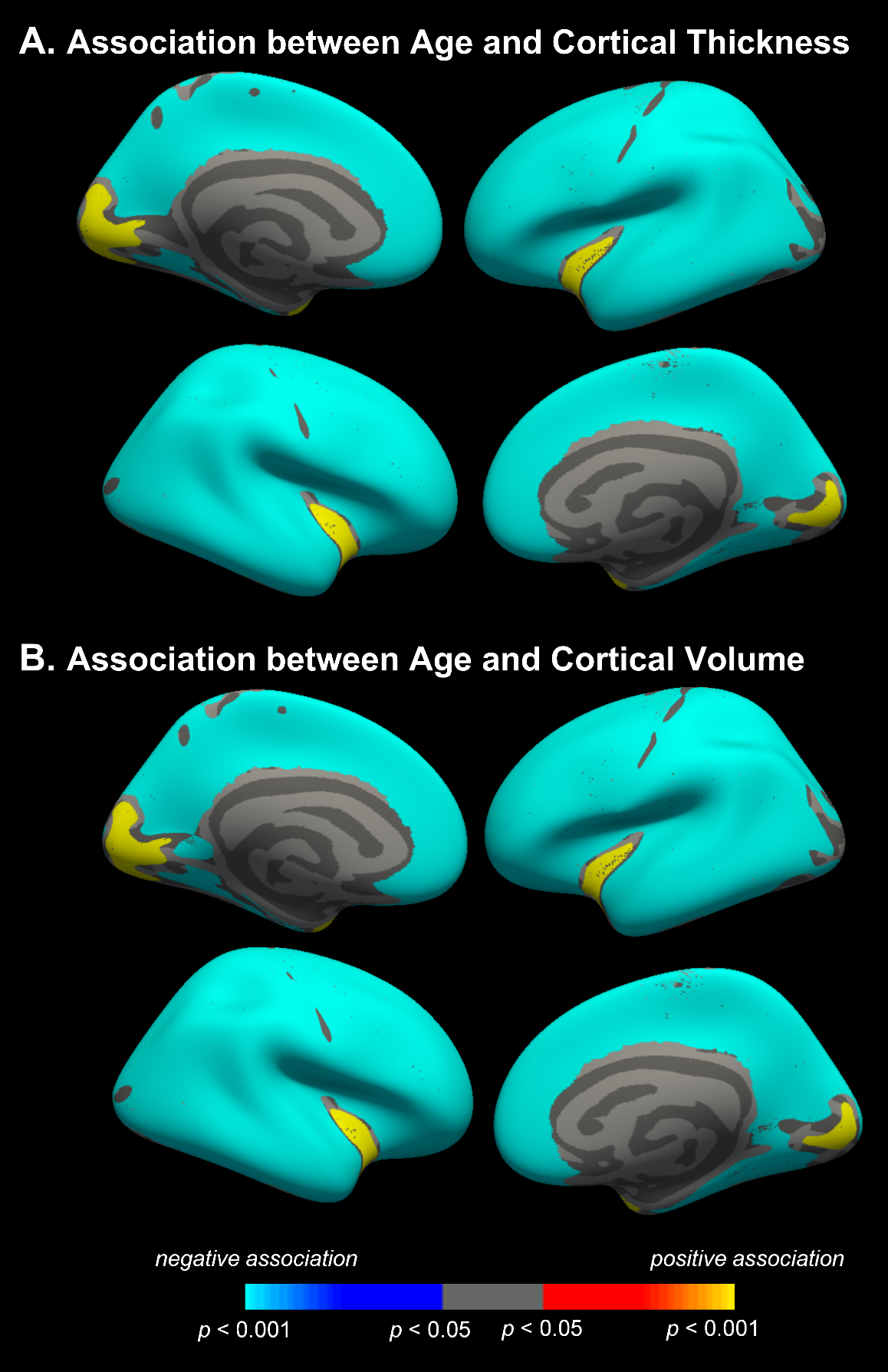
**
